# Supplementary material for: Causal interplay between lactose intolerance and gut microbiota: a combined bidirectional Mendelian randomization and in vivo validation study
Source: Front Nutr. 2026 Jun 1;13:1803337. doi: 10.3389/fnut.2026.1803337 (PMC13265576; doi:10.3389/fnut.2026.1803337)
Supplement: Supplementary file 1 [file Data_Sheet_1.zip › supplementary materials/Forward/leave one out cross validation/ebi-a-GCST90027478.finngen_R12_E4_LACTONAS.pdf]

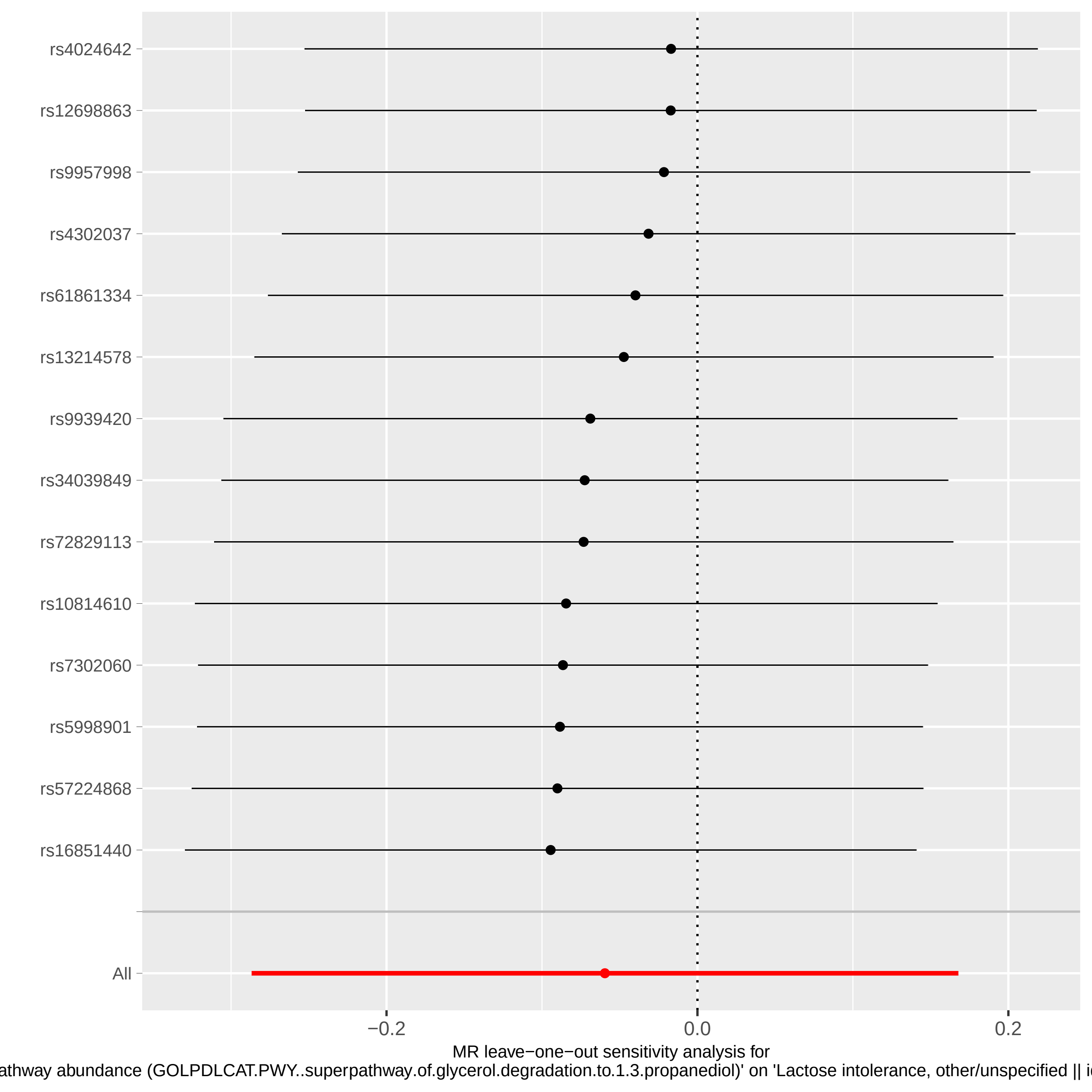
rs4024642
rs12698863
rs9957998
rs4302037
rs61861334
rs13214578
rs9939420
rs34039849
rs72829113
rs10814610
rs7302060
rs5998901
rs57224868
rs16851440
All
−0.2
0.0
0.2
MR leave−one−out sensitivity analysis for
athway abundance (GOLPDLCAT.PWY..superpathway.of.glycerol.degradation.to.1.3.propanediol)' on 'Lactose intolerance, other/unspecified || i
